# Harnessing BiOI/V_2_O_5_ Nanocomposites: Advanced Bifunctional Catalysts for Visible-Light Driven Environmental Remediation and Antibacterial Activity

**DOI:** 10.3390/molecules30122500

**Published:** 2025-06-06

**Authors:** Anil Pandey, Narayan Gyawali, Devendra Shrestha, Insup Lee, Santu Shrestha, Subas Acharya, Pujan Nepal, Binod Gaire, Vince Fualo, Sabita Devi Sharma, Jae Ryang Hahn

**Affiliations:** 1 Department of Chemistry, Jeonbuk National University, Jeonju 54896, Republic of Korea; pandeyan2001@gmail.com (A.P.); naran.gywli@gmail.com (N.G.); ads6846@gmail.com (I.L.); santu_shrestha@hotmail.com (S.S.); link2subas@gmail.com (S.A.); pujannepal25@gmail.com (P.N.); gairebinod@gmail.com (B.G.); fualo.vc@gmail.com (V.F.); 2 Department of Bionanotechnology and Bioconvergence Engineering, Graduate School, Jeonbuk National University, Jeonju 561-756, Republic of Korea; 3 Department of Physics, Birendra Multiple Campus, Chitwan 44207, Nepal; sabitapaudel086@gmail.com; 4 Textile Engineering, Chemistry and Science, North Carolina State University, 2401 Research Dr., Raleigh, NC 27695-8301, USA

**Keywords:** BiOI/V_2_O_5_ composite, visible-light-driven photocatalysts, *p-n* heterojunction, antibacterial activity, wastewater treatment

## Abstract

Efficient photocatalysts based on composite materials are essential for addressing environmental pollution and enhancing water purification. This study presents a novel BiOI/V_2_O_5_ nanocomposite (BVNC) with a flower-like layered structure, synthesized via a low-temperature solvothermal process followed by high-pressure annealing for visible light (VL)-driven dye degradation and antibacterial activities. Compared to individual BiOI nanoparticles (BOINP) and V_2_O_5_ nanoparticles (VONP), under VL, the BVNC demonstrated significantly enhanced photocatalytic and antibacterial activity. The best-performing BVNC achieved a remarkable methylene blue degradation efficiency of 95.7% within 140 min, with a rate constant value 439% and 430% of those of BOINP and VONP, respectively. Additionally, BVNC exhibited high photocatalytic efficiencies for rhodamine 6G (94.0%), methyl orange (90.4%), and bisphenol A (69.5%) over 160 min, highlighting the superior performance of the composite materials for cationic and anionic dyes. Furthermore, BVNC established outstanding antibacterial capability against *Staphylococcus aureus* and *Escherichia coli,* demonstrating zones of inhibition of 12.24 and 11.62 mm, respectively. The improved catalytic and antibacterial capability is ascribed to the presence of a robust *p-n* heterojunction between BOINP and VONP, which broadens the photo-absorption range, reduces bandgap energy, and facilitates the significant separation of excitons and faster release of reactive oxygen species.

## 1. Introduction

Water is an essential resource for all living organisms, yet its quality is increasingly threatened by industrial contamination. The rapid expansion of chemical industries, particularly those involved in textiles, paints, leather, pesticides, food, and cosmetics, has introduced a wide range of pollutants into water sources, posing severe risks to human health and ecosystems. These industries discharge synthetic organic and inorganic compounds, including toxic trace metal ions, into wastewater and the environment [1,2]. Among these contaminants, cationic dyes such as methylene blue (MB) and rhodamine 6G (R6G), as well as the anionic dyes methyl orange (MO), are particularly concerning due to their harmful effects on human health, including headaches, mental confusion, and nausea [3,4]. Most importantly, bisphenol-A (BPA), which is widely found in plastic bottles, food packaging, and even the linings of metal cans, is a persistent environmental toxin recognized to disrupt the endocrine system and has been linked to cancer, infertility, and other health issues [5,6]. Therefore, to safeguard public health and preserve ecosystems, effective wastewater treatment technology is highly desirable. Among various wastewater treatment techniques [7,8], photocatalysis has emerged as a highly effective and environmentally friendly approach. In the presence of light, this technique utilizes reactive species, such as hydroxyl radicals, peroxide radicals, and holes, to break down organic pollutants into harmless inorganic byproducts, thereby ensuring cleaner and safer water [9,10]. However, designing robust photocatalysts that harness the optimal sunlight to convert solar energy into chemical energy still remains challenging. Therefore, the synthesis of a novel semiconductor-based composite material composed of BiOX and V₂O₅ may serve as a promising alternative to conventional V₂O₅-based photocatalysts.

BiOX (X = Cl, Br, I) has been developed as a highly potential photocatalyst because of its unique layered structure, ensuring the exposure of higher active sites, and tunable bandgap, which enables efficient light absorption and charge transport [11]. Among these, BiOI stands out with the narrowest bandgap (1.6–1.9 eV), allowing for superior visible light utilization. Additionally, its layered structure, coupled with abundant structural defects, enhances photocatalytic activity by providing active sites for pollutant degradation [12]. However, despite these advantages, BiOI experiences the quick excitons (electron–hole) recombination, which significantly challenges its photocatalytic efficiency [13]. To address this limitation, researchers have explored various strategies, such as heterojunction formation, ion doping, and morphological control, to extend charge carrier lifetimes and enhance photocatalytic performance. Among these approaches, heterojunction engineering has proven to be particularly effective. For instance, Cheng et al. [14] synthesized AgI/BiOI composites using a simple, low-temperature chemical bath method, which exhibited significantly improved photocatalytic efficiency in decomposing pollutants such as methyl orange and phenol. This improvement is attributed to the efficient retention and transport of carriers at the AgI/BiOI interface. Recent studies have further advanced on BiOI-based heterostructures by incorporating materials such as TiO_2_ [15], g-C_3_N_4_ [16], ZnO [17], and α-Fe_2_O_3_ [18].

Based on our knowledge, no study has reported the BiOI composited with vanadium pentoxide (V_2_O_3_); however, it has been a popular material for making composites. For instance, Su et al. [19] successfully synthesized microporous V_2_O_5_/BiVO_4_ composites using colloidal carbon spheres as a template, forming an effective heterojunction that significantly improved charge carrier separation and lifetime, resulting in improved photocatalytic performance. Additionally, various V_2_O_5_-based binary heterostructures, such as V_2_O_5_/ZnO [20,21], V_2_O_5_/g-C_3_N_4_ [22], and SnO_2_/V_2_O_5_ [23], have demonstrated improved not only the photocatalytic but also the antibacterial performance. Therefore, we hypothesize that our as-synthesized novel composite may exhibit improved performance in photocatalytic and antibacterial applications as a bifunctional catalyst.

The global microbial population plays a critical role in shaping ecosystems, supporting human health, and environmental balance. This population is rapidly expanding due to factors such as increased food availability and habitat expansion [24]. While many microorganisms contribute to essential ecological functions, a significant number are pathogenic, posing serious threats to human health and the stability of ecosystems [25]. Among them, Gram-positive *Staphylococcus aureus* (*S. aureus*) and Gram-negative *Escherichia coli* (*E. coli*) are responsible for a wide range of infections, from minor skin conditions to life-threatening ones such as pneumonia and urinary tract infections [25]. The growing prevalence of these harmful pathogens underscores the urgent need for effective antibacterial strategies. Researchers are actively developing innovative antibacterial materials that are not only highly efficient but also biocompatible, cost-effective, and environmentally friendly [26,27].

Herein, this study reports a novel composite of BiOI/V_2_O_5_ featuring *p-n* heterojunctions, synthesized via a solvothermal process followed by high-temperature and high-pressure annealing. Effective formation of *p-n* heterojunction and favorable band alignment between BiOI and V_2_O_5_ promote the driving of redox reactions, overcoming prompt recombination of electron–hole pairs and the slow production of reactive oxygen species (ROS) problems. Notably, the BVNC demonstrated superior photocatalytic efficiency to decompose a range of organic pollutants—from cationic to anionic—including MB, R6G, MO, and BPA. In addition to the pH-dependent investigation, studies on pollutant concentration and catalyst dosage were conducted to determine the optimal conditions for photocatalytic performance. Additionally, the composite exhibited robust antibacterial activity and maintained its structural integrity and photocatalytic efficiency after multiple cycles. Therefore, the bifunctionality properties of our composite may allow it to stand out on its own from the conventional materials and become a benchmark for further improvement.

## 2. Results and Discussion

### 2.1. Morphological and Physicochemical Characterization

**Figure 1:**
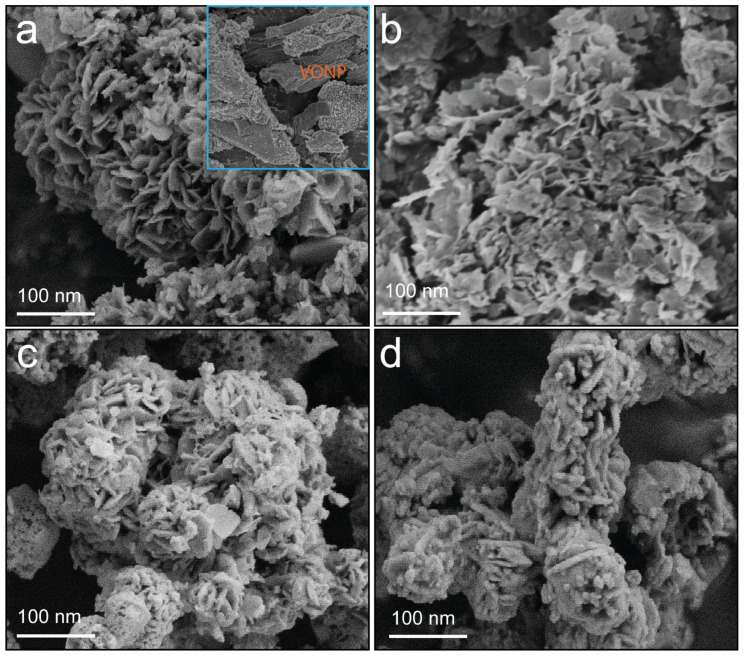
FE-SEM images of various nanoparticles and nanocomposites: (**a**) flower-like BOINP, (**b**) as-synthesized nanocomposite BVNC-1, (**c**) BVNC-2, and (**d**) BVNC-3. The inset in (**a**) displays the FE-SEM image of VONP.

The morphology of BOINP, VONP, and BVNC was analyzed using FE-SEM. Individual BiOI nanoparticles exhibited a unique structure composed of thin nanoplates, as shown in Figure 1a. The VONP features a thin nanosheet architecture (inset of Figure 1a). The addition of V_2_O_5_ precursors has slightly modified the BiOI morphology, producing smaller, compact layered structures. At lower V_2_O_5_ concentrations, the BiOI nanoplates became thinner, with randomly dispersed VONP nanosheets on their surfaces (Figure 1b). In contrast, at higher V_2_O_5_ precursor concentrations, the BiOI structure is obscured, which is attributed to a V_2_O_5_ coating (Figure 1c,d). The presence of respective elements in the composite was further supported by the EDS color mapping analysis of BVNC-1, as disclosed in Figure S1. The EDS results indicated the following atomic percentages in the composite: Bi (64.4%), V (5.9%), I (12.2%), and O (17.5%). The results are in good agreement with the theoretical calculation (Table S1). FE-SEM-EDS analysis revealed the development of a well-defined heterojunction in the BVNC, with VONP nanosheets effectively anchored on the flower-like surface of BiOI nanoplates. This physical junction between the semiconductors is expected to enhance the formation and segregation of photo-induced excitons through the *p-n*-type heterostructure of the nanocomposites, leading to improved photocatalytic performance.

**Figure 2:**
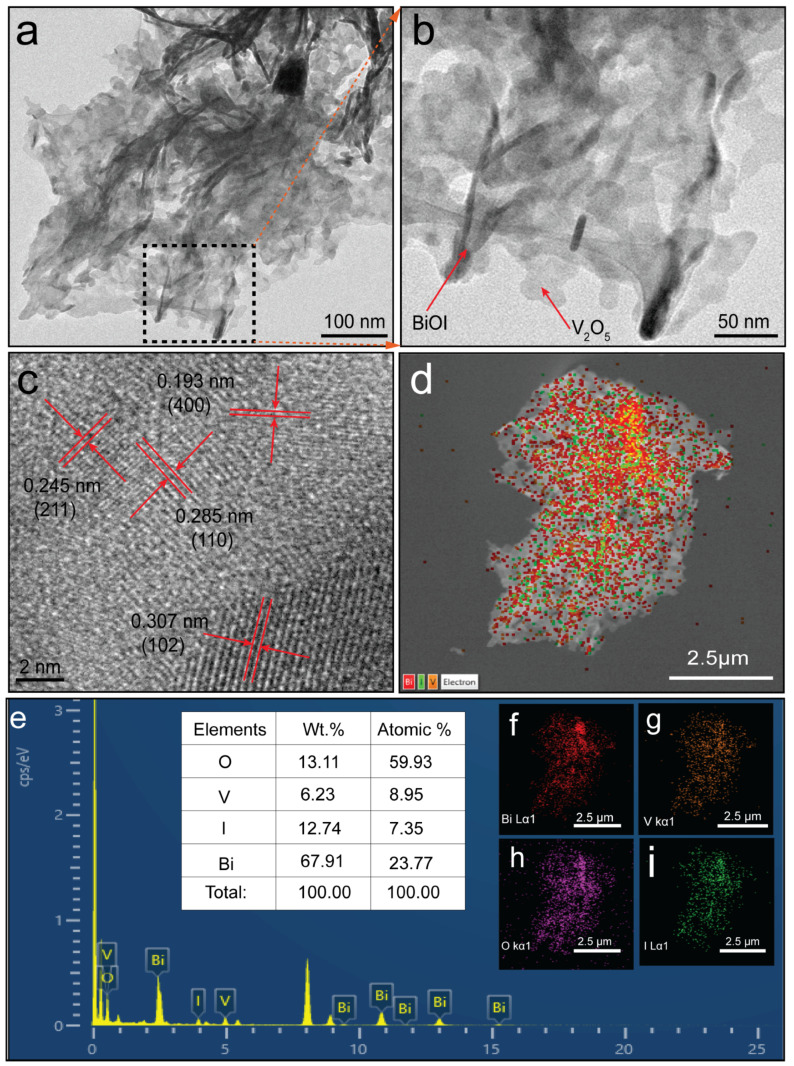
Low (**a**) and high resolution (**b**) HR-TEM images of BVNC-1; lattice fringe (**c**), EDS for elemental color mapping (**d**) and EDS spectrum of the whole region of figure (**e**) of Bi (**f**), V (**g**), O (**h**), I (**i**), respectively, in BVNC-1.

**Figure 3:**
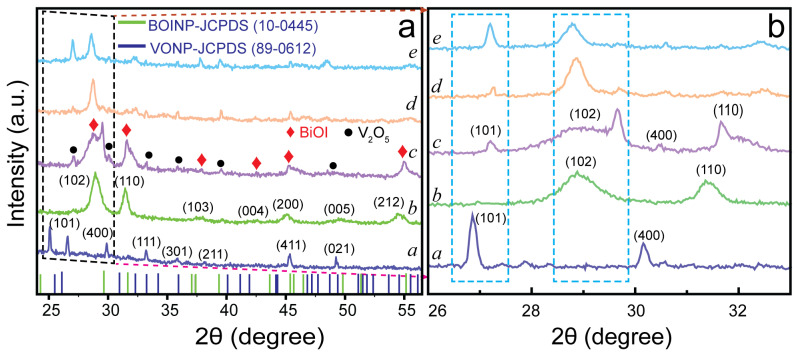
HR-XRD peaks (**a**) of as-synthesized photocatalysts: (curve *a*) VONP, (curve *b*) BOINP, (curve *c*) BVNC-1, (curve *d*) BVNC-2, and (curve *e*) BVNC-3, and (**b**) magnified XRD patterns.

Figure 2 displays HR-TEM analysis of BVNC-1, where over the BOINP nanoflakes, the sheet-like thin layers of VONP are uniformly dispersed across the flakes. The lattice fringes 0.307 and 0.285 nm calculated from HR-TEM images (Figure 2c) correspond to 102 and 110 planes of BiOI. Similarly, 0.220 and 0.193 nm agree with 211 and 400 planes of V_2_O_5_; the result is further varied by XRD analysis (Figure 3a,b). Aligned with SEM-EDS, the TEM-EDS analysis further confirmed the elemental make-up of the as-synthesized materials, showing the presence of Bi, I, O, and V, as illustrated in Figure 2d–i. The corresponding spectra revealed atomic percentages of 67.9% for Bi, 6.2% for I, 12.7% for O, and 13.1% for V.

The crystalline properties of the individual nanoparticles and the as-synthesized composites were observed using XRD plots, as indicated in Figure 3. From the results of the experiment, the main diffractions of VONP at 2θ angles of 26.57°, 29.87°, 33.21°, 35.81°, 45.35°, and 49.56° were indexed to the (101), (400), (111), (221), (411), and (021) planes, respectively, as shown in Figure 3a (curve *a*). These peaks confirm the orthorhombic crystalline phase of VONP, which is consistent with the reference pattern from the JCPDS card No. 89-0612 [28,29]. Similarly, the main diffraction peaks at 28.99°, 31.50°, 45.14°, and 54.77° were ascribed to the (102), (110), (200), and (212) crystallographic facets of BiOI, respectively, as displayed in Figure 3a (curve *b*). These peaks confirm the formation of the tetragonal phase of BiOI, and are in agreement with JCPDS card 10-0445 [30].

When V_2_O_5_ was loaded onto BiOI, a slight decrease in peak intensity, peak broadening, and a shift towards higher angles were observed (Figure 3a, curves *c*–*e*). These changes are likely due to strong chemical interactions between the nanoparticles. Additionally, other factors such as elemental interactions between V_2_O_5_ and BiOI, partial redox reactions affecting the oxidation state of vanadium, or structural rearrangements at the interface may also contribute to the observed modifications in the diffraction pattern.

By using Scherrer’s formula (*d* = 0.9 *λ*/βcos*θ*, where d, *λ*, β, and *θ* represents the crystallite size, wavelength, full width at half maximum of the most intense peak, and the Bragg angle of the crystal, respectively), the crystallite size of the as-synthesized composites was examined. The average crystallite sizes of BVNC-1, BVNC-2, and BVNC-3, as estimated using Gaussian fitting of the most intense (102) peak, are approximately 39, 37, and 35 nm, respectively. The magnified XRD patterns revealed peak shifts towards higher angles in the hetero-composites. However, the persistence of the characteristic peaks of BOINP and VONP in the composite materials indicates that the crystalline structure of the nanoparticles remained intact in the synthesized heterojunctions (Figure 3b).

**Figure 4:**
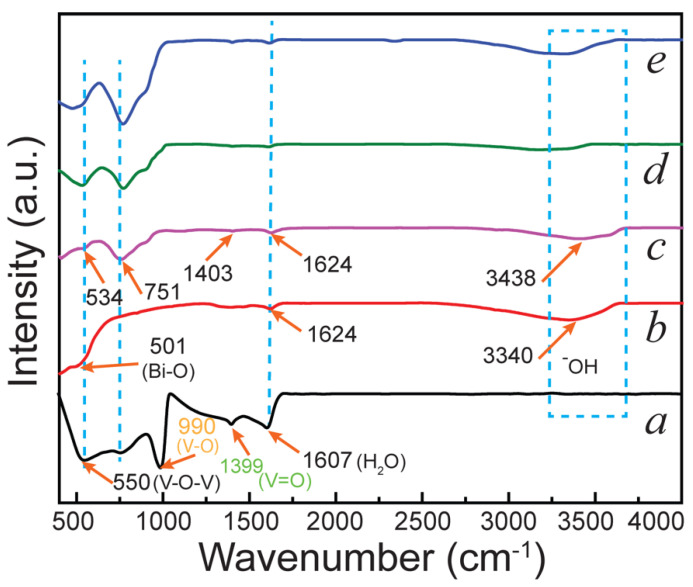
FT-IR spectra of VONP (curve *a*), BOINP (curve *b*), BVNC-1 (curve *c*), BVNC-2 (curve *d*), and BVNC-3 (curve *e*), respectively.

FT-IR spectroscopy was used to analyze the surface groups of both the individual components and the synthesized composite materials. The FT-IR spectra of VONP, BOINP, and BVNC samples, measured in the 400–4000 cm⁻^1^ range via the KBr-method, are displayed in Figure 4. The curve of VONP (Figure 4, curve *a*) exhibits distinct peaks at 1399, 990, and 550 cm⁻^1^ corresponding to the stretching of V = O, V-O, and V-O-V, respectively [31]. A distinct band at 501 cm⁻^1^ confirms the characteristic peak of BOINP, associated with the stretching mode of the Bi-O bond [32]. The appearance of new peaks at 534 and 751 cm⁻^1^ attributed to the stretching and angular vibrations of Bi-O-V bonds, respectively, confirms the successful formation of Bi-O-V linkages within the hetero-composite structures.

Additionally, a bending vibration around 1607 and an absorption band around 3340 cm⁻^1^ (Figure 4(a–e)) were due to free water molecules and hydroxyl groups [33]. These results indicate strong chemical interactions between BiOI and V_2_O_5_ nanoparticles, ensuring the effective integration of VONP onto the BOINP surface and the creation of the intended heterojunction interface. Raman analysis (Figure S2) further demonstrated the presence of the different bonds in the respective samples. The VONP exhibits multiple peaks, which are located at 144, 197, 330, 622, 801, and 980 cm^−1^. The peak at 144 cm^−1^ corresponds to the skeleton bent vibration (B_3g_ mode). The bending vibration of O–V–O corresponds to the peak at 197 cm^−1^, while the peak at 330 cm^−1^ corresponds to the oscillating mode of V–O. The peaks at 622 and 801 cm^−1^ are due to the vibration mode of (V–O_3_–V), respectively [34]. Furthermore, the peak at 980 cm^−1^ corresponds to stretching of V = O, which signifies the layer-type structure of V_2_O_5_ [35]. However, Raman broad bands appear at 120 and 157 cm^−1^ for the BiOI single component, corresponding to the external A_1g_ Bi–I and internal E_1g_ Bi-I stretching mode, respectively [36]. However, the peak at 307 cm^−1^ is due to the energy supplied locally by the laser irradiation during the experimentation [37]. The shift in the respective peaks on VONP and BOINP on BVNC-1, -2, and -3 is attributed to the formation of the Bi-O-V bond in the composites and their chemical interaction.

**Figure 5:**
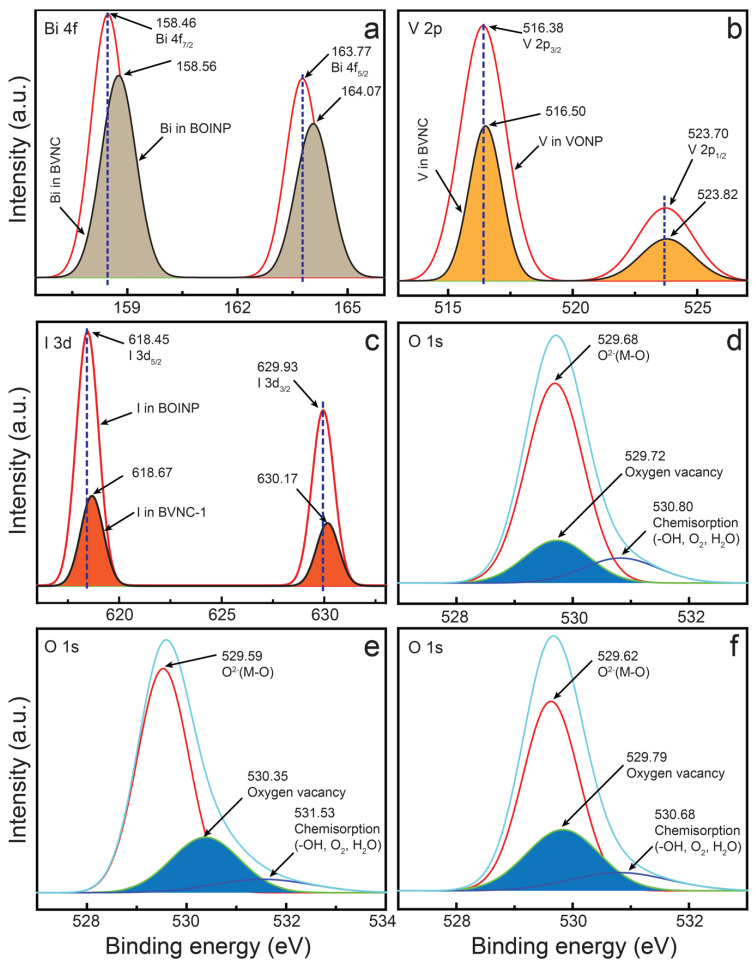
HP-XPS comparative spectra of (**a**) Bi-4f7/2 and 4f5/2 spectra of BVNC−1 composite (red line) and individual BOINP (black line), (**b**) V-2p spectra of the BVNC-1 composite (red line) and individual VONP (black line), (**c**) I 3d spectra of the BVNC-1 composite (red line) and individual BOINP (black line), and O 1s core−level spectrum of BVNC-1 (**d**), BVNC-2 (**e**), and BVNC-3 (**f**), respectively.

Figure 5 and Figure S3 display the HR-XPS analysis to identify the chemical environment in BOINP, VONP, and BVNC samples. Figure 5a revealed Bi orbital splitting into the 4*f*_7/2_ and 4*f*_5/2_ with a difference of 5 eV, which demonstrated the existence of the trivalent state of Bi [32]. For the composite, BVNC-1, the respective peaks are shifted slightly toward higher binding energy. Similarly, the V-2p spectrum displayed distinct orbital splitting of the peaks into the 2p_3/2_ and 2p_1/2_ corresponding to the pentavalent state of V [38], while the respective peak shift observed in BVNC-1 compared to uncombined V_2_O_5_ (Figure 5b). The I-3d spectrum showed strong peaks attributed to I⁻ in BiOI [39], with positive shifts observed in BVNC-1 compared to uncombined BiOI, as shown in Figure 5c. The peaks’ positions around 529, 530, and 531 eV (Figure 5d,e) for O-1s represent the presence of a bond between metal (Bi or V) and oxygen, lattice oxygen, adsorbed O_2_, H_2_O, and –OH groups in BVNC-2 and BVNC-3, respectively. The characterization techniques’ results demonstrated the successful creation of hetero-junctions and chemical interactions between BOINP and VONP, suggesting the potential to improve charge segregation and transfer efficacy, thereby boosting photocatalytic activity.

### 2.2. Optical Properties: Photoluminescence and Bandgap Evaluations

Photoluminescence (PL) analysis was used to investigate retention of photo-induced electron–hole recombination in BVNC heterostructures (Figure S4). Individual BOINP and VONP displayed emission bands at 417, 450, and 481 nm. The emission peak near 400 nm originates from the radiative recombination of free excitons transitioning from the V-3d conduction band minimum to the O-2p valence band maximum. Additionally, the recombination of excitons from the split-off V-3d conduction band to the top of the O-2p valence band contributes to the PL emission peak observed around 450 nm [40]. Compared to BIONP, VONP shows a weaker intensity, indicating better charge separation. The PL intensity gradually decreased with increasing V_2_O_5_ compared to BOINP. BVNC-1 exhibited the lowest intensity, confirming effective heterostructure formation and suitable band edge alignment. The presence of heterojunctions inhibited the recombination of photogenerated carriers, resulting in enhanced performance due to greater availability of carriers for redox processes under visible light irradiation [41].

The band gap is a crucial factor in photocatalysis, as it influences electron–hole pair generation and photocatalyst performance. The optical band gaps of BOINP, VONP, BVNC-1, BVNC-2, and BVNC-3 were determined using UV-Vis spectroscopy in conjunction with the Tauc equation with modification [42]. The modified Tauc equation is:

(*α* (*hν*) (*hν*))^1/n^ = *A* (*hν* − *E*_g_) (1)

**Figure 6:**
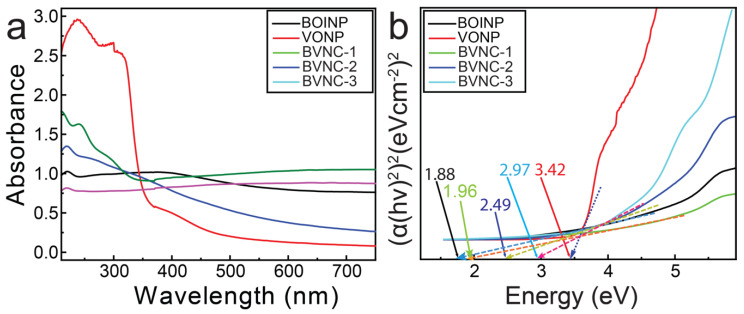
UV-Vis absorbance curves (**a**) and Tauc plots (**b**) illustrating the optical bandgap of BOINP, VONP, BVNC-1, BVNC-2, and BVNC-3, respectively.

Here, *n* = 1/2 (for direct transition), *α* denotes the absorption coefficient, *A* is a constant, *h* is Planck’s constant, *E*_g_ represents the bandgap, and *v* represents the photon frequency. The UV-Vis spectra of the individual and composite nanoparticles are presented in Figure 6a, while the corresponding Tauc plots are depicted in Figure 6b. The bandgap was determined by plotting the Tauc plot, in which (*α*(*hν*)^2^)^2^ was plotted against photon energy, and the bandgap value was determined by extrapolating the linear portion of the plot to the X-axis. The band gaps were calculated as 1.88, 3.42, 1.96, 2.49, and 2.97 eV for BOINP, VONP, BVNC-1, BVNC-2, and BVNC-3, respectively. It is noted that the values for individual BOINP and VONP are in accordance with the reported values of BiOI and V_2_O_5_ [12,43,44]. The notable reduction in bandgap from 3.42 to 1.96 eV for BVNC-1 is due to the successful production of a well-defined heterojunction between BOINP and VONP, as corroborated by FTIR and TEM analyses. Therefore, BVNC-1 exhibited strong absorption across the explored wavelength range, with red-shifted absorption peaks indicating enhanced VL harvesting and increased generation of excess electron–hole pairs, which are essential for the catalytic decomposition of dyes.

### 2.3. Adsorption and Photocatalytic Degradation Performance

**Figure 7:**
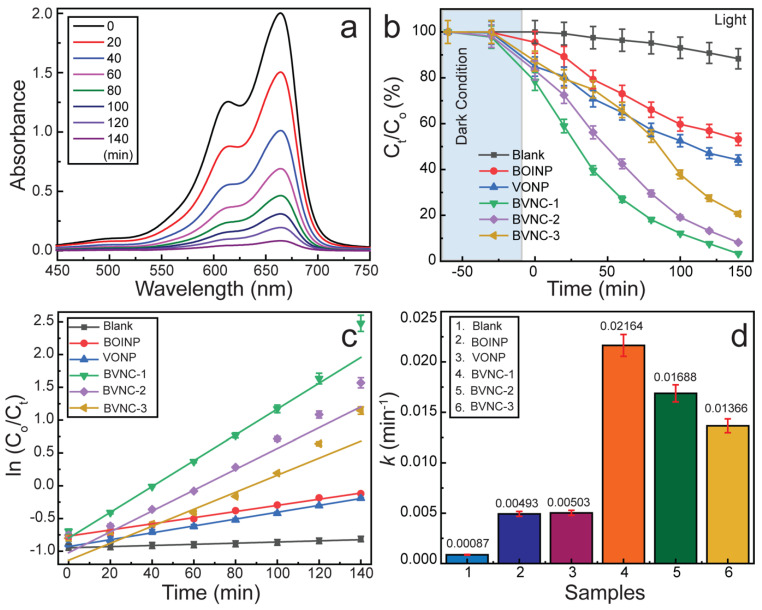
Absorbance spectra of BVNC-1 (**a**), comparative photocatalytic performance (**b**), kinetic plot (**c**), and rate constant (**d**) for decomposition of MB in the presence of Blank, BOINP, VONP, BVNC-1, BNVC-2, and BVNC-3, respectively.

The adsorption and photocatalytic efficiency of the samples was assessed by degrading 15.0 mg/L of MB under VL irradiation. UV-Vis spectroscopy was used to understand the photo-degradation behavior of methylene blue, and their corresponding spectra are displayed in Figure 7a and Figure S5a–e. Degradation efficiencies of MB were evaluated for five different photocatalysts: BOINP, VONP, BVNC-1, BVNC-2, and BVNC-3. A Blank test (without photocatalysts) was performed. The purpose of the Blank test was to observe the impact of the MB, as it is a good photosensitizer, and other experimental setups in the degradation of dyes by the as-synthesized photocatalysts. The efficiencies observed under VL irradiation for 140 min were 48.2%, 44.5%, 95.7%, 90.1%, and 85.6%, respectively. BVNC-1 demonstrated the greatest degradation efficiency for aqueous solutions of the MB solution. A pseudo-first-order kinetic model was applied to analyze the decomposition of MB by photocatalysts, with all samples exhibiting correlation coefficients (R^2^) exceeding 0.95, as shown in Table S2 (Supplementary Materials). The pseudo-first-order kinetics of MB degradation were confirmed by linear curves of ln (C₀/Ct) vs. t (Figure 7b,c)**,** and the rate constant (*k*) was estimated using Equation (6). BVNC-1 demonstrated the highest rate constant for the degradation of MB with values of 0.02164, while the Blank test, BOINP, VONP, BVNC-2, and BVNC-3 have values of 0.00087, 0.00493, 0.00503, 0.01688, and 0.01366 min⁻^1^, respectively. For the Blank test, 11.4% of MB degradation occurred during VL irradiation for 140 min (Figure S5f); therefore, the contributions of MB and other experimental sets to the overall performance of the photocatalysts are minimal. Along with photocatalytic activity, we have presented the adsorption behavior of as-synthesized materials in Figure 7b. The results showed that the best-performing material demonstrated the highest adsorption capacity compared to the others.

Similarly, the best-performing photocatalyst, BVNC-1, was further employed to evaluate its photocatalytic efficiency in the decomposition of R6G, MO, and BPA under similar experimental conditions. Within 160 min, 93.9% of R6G, 90.4% of MO, and 69.5% of BPA were degraded. The photocatalytic degradation efficiencies of R6G, MO, and BPA, along with the kinetic plot, R^2^, and rate constant values, are shown in Figure S6. Although a pseudo-first-order model is commonly employed for photocatalytic degradation studies, the fitting results (Figure S6d) suggest that a zero-order kinetic model better describes the degradation behavior of MO by BVNC-1, as indicated by higher R^2^ (0.99) values and improved linearity. This implies that surface-limited processes may dominate the reaction kinetics for these samples under our experimental conditions. The BVNC-1 heterojunction composite exhibited outstanding degradation performance for organic pollutants compared to previously reported photocatalysts (Table S3).

### 2.4. Effect of Catalyst Dose, Pollutant Dose, and Reusability

For the optimized performance of the BVNC-1 photocatalyst in removing MB, we studied the effect of catalyst and pollutant doses. The catalyst dosage was varied from 0.3 g/L to 0.7 g/L. As the catalyst dosage was raised from 0.3 to 0.5 g/L, it led to an enhancement in the decomposition efficiency of MB, rising from 80.1% to 95.7% (Figure S7a). This improvement was mainly due to the enhanced adsorption capacity and more active sites available for photocatalytic reactions. However, when the catalyst dose exceeded beyond 0.5 g/L to 0.7 g/L, the decomposition efficiency declined. This decline was attributed to reduced light penetration, which hindered the excitation of photocatalyst particles, thus lowering photocatalytic performance. The optimal catalyst dose, which resulted in the highest degradation efficiency, was determined to be 0.5 g/L.

Similarly, the influence of MB concentration (10.0, 15.0, and 20.0 mg/L) on the BVNC-1 catalyst demonstrated that removal efficiencies were recorded as 87.3%, 95.7%, and 94.0% for the respective concentrations (Figure S7b). At lower concentrations, there were limited interactions between the pollutant and the catalyst, which resulted in reduced efficiency. At an optimal concentration of 15.0 mg/L, the increased interaction between the pollutant and photocatalyst led to enhanced generation of active species [45]. However, at the highest concentration (20.0 mg/L), pollutant accumulation on the catalyst restricted access to active sites, thereby reducing the generation of active species [45,46]. Also, at higher concentrations, the light penetration is content, which ultimately limits absorption of photons by the catalyst [47].

**Figure 8:**
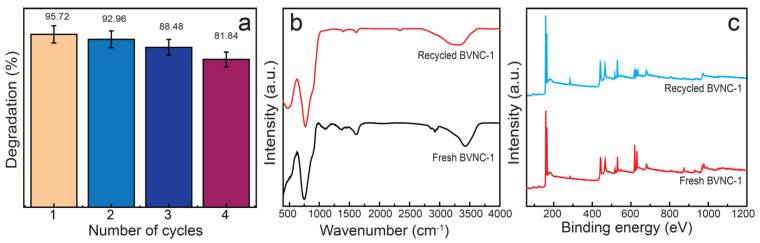
Recyclability tests of BVNC-1 (**a**), FT-IR spectra (**b**), and XPS survey plots from XPS of fresh and recycled BVNC-1 (**c**).

The recyclability of BVNC-1 was tested over four cycles, with the catalyst maintaining a consistent MB degradation performance of over 80%. A minor reduction in activity was noted following the fourth cycle (Figure 8a). The stability of the catalyst was confirmed by FT-IR and XPS after four reuse cycles, with all spectral peaks remaining unchanged (Figure 8b,c). Therefore, the composite is robust and may be industrially applicable as an alternative photocatalyst.

### 2.5. Effect of Point of Zero Charge and pH

The point of zero charge (PZC) refers to the pH at which the catalyst surface has no net charge, meaning the surface charge is balanced. In photocatalysis, pH is a critical element that affects both the adsorption of dyes on the catalyst surface and their decomposition efficiency. At a pH below PZC, the surface of the catalyst acquires a positive charge, which promotes the electrostatic attraction of negatively charged particles, such as anionic dye molecules. Conversely, when the pH exceeds PZC, the surface of the catalyst obtains a negative charge, facilitating the adsorption of positively charged particles. Maintaining the solution pH close to the PZC can influence adsorption and catalytic efficiency, depending on the specific interactions between the catalyst surface and the molecules.

To perform the experiment, solutions with pH values ranging from 2 to 12 (2, 4, 6, 8, 10, and 12) were prepared in a series of vials by adjusting the pH to the desired initial value (pH_initial), adding 0.01 M HCl or 0.01 M NaOH. Next, 10.0 mg of photocatalyst was added to each vial. After 24 h of continuous stirring, the final pH was measured. To determine the PZC, the difference between the initial and final pH was plotted against the initial pH, as shown in Figure S8. The PZC of BVNC-1 was found to be 6.77, which is close to the neutral pH of 7. This near-neutral PZC enhances the adsorption of both positively and negatively charged dyes onto the photocatalyst, thus improving photocatalytic efficiency [48]. Furthermore, we conducted a pH-dependent study for both the cationic dye, MB, and the anionic dye, MO (Figure S9). The results show that for the cationic dye, higher photocatalytic activity was observed at higher pH values. In contrast, for the anionic dye, the degradation percentage was highest at lower pH levels, which can be attributed to the pollutant’s charge-dependent interactions.

### 2.6. Active Charge Trapping Test

To evaluate the contribution of reactive species in the photocatalytic reaction of MB under VL illumination, scavenger tests were conducted employing IPA, BQ, and KI as trapping agents for ^•^OH, O_2_^•−^, and h^+^, respectively. The results of these tests, evaluating the degradation of MB by BVNC-1 with each individual scavenger, are shown in Figure S10. Without any scavengers, the photocatalytic degradation efficiency of MB reached 95.7%. However, when IPA was introduced to the reaction, the decomposition efficiency reduced to 80.3%, suggesting that •OH has a minor role in MB decomposition. The addition of KI resulted in a further reduction, with MB degradation dropping to 58.9%, highlighting the role of holes in promoting the degradation reaction. Similarly, the BQ addition to the system had the most substantial inhibitory effect, reducing MB degradation to only 25.4%. These findings indicate that ^•^OH, O_2_^•−^, and h^+^ all facilitate significantly the photodegradation. Among these species, O_2_^•−^ appears to be the most dominant, as BQ had the most pronounced inhibitory effect. The effectiveness of the scavengers can be ranked as BQ > KI > IPA, and the sequence of active species is identified as O_2_^•−^ > h^+^ > ^•^OH. While the role of photogenerated holes was comparatively minor, the involvement of hydroxyl radicals had a notable impact on enhancing the reaction rate. Therefore, all three reactive species were generated and played a significant role in promoting the photocatalytic decomposition pathway [1].

### 2.7. Proposed Photocatalytic Mechanism

When exposed to VL, electrons in the photocatalyst’s valence band (VB) become excited to the conduction band (CB), thereby initiating the photocatalytic reaction [49]. The excited electrons in the conduction band serve as reducing agents [50], whereas the holes remaining in the valence band function as oxidizing agents (Equation (2)).

hν + photocatalyst → (e^−^_CB_ + h^+^_VB_) photocatalyst (2)

The edge potential of the VB and CB is a key factor in determining the reaction mechanism. These potentials are determined using Equations (3) and (4):

*E*_CB_ = χ − *E*_e_ − 0.5 *E*_g_ (3)

*E*_VB_ = *E*_CB_ + *E*_g_ (4)

In these equations, *E*_e_ represents the free-electron energy level, typically taken as 4.5 eV, the electronegativity of the semiconductor is represented by χ (χ = 5.94 eV for BiOI [51], χ = 6.10 eV for V_2_O_5_ [52]), *E*_VB_ and *E*_CB_ represent the VB and CB edge potential [53], respectively. *E*_g_ represents the optical energy bandgap of BiOI and V_2_O_5_, with the corresponding values of 1.88 eV and 3.42 eV, respectively, as determined from the Tauc plot Figure 6b. Based on Mulliken electronegativity theory, the calculated values of the *E*_CB_ and *E*_VB_ for BiOI and V_2_O_5_ are +0.087 eV, −0.523 eV, +1.967 eV, and +2.897 eV vs. the normal hydrogen electrode at pH 7, respectively.

**Scheme 1:**
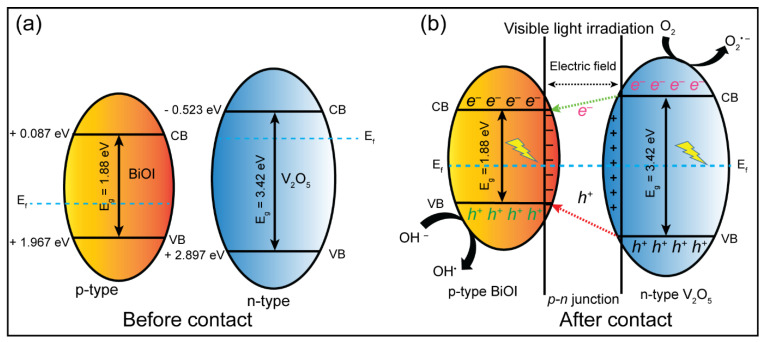
Scheme of the energy band of (**a**) *p*-BiOI and *n*-V_2_O_5_ before contact, and (**b**) the formation of the *p*-*n* junction of *p*-BiOI to *n*-V_2_O_5_ under VL illumination.

Based on the experimental data, the conduction band potential of V_2_O_5_ (−0.523 eV) is more negative than the standard reduction potential (−0.33 eV) required to convert O_2_ into O_2_^•−^, which means that it is sufficient to directly produce superoxide radicals. Therefore, as shown in Scheme 1, the peroxide radicals can be generated on the surface of VONP, while the hydroxyl radicals are generated on the surface of BIONP; thus, we propose a *p-n* junction mechanism to explain the photocatalytic efficiency of the BiOI/V_2_O_5_ heterojunction catalyst. BiOI acts as a *p*-type semiconductor, with its Fermi energy level (*E*_f_) positioned near the VB, while V_2_O_5_ functions as an *n*-type semiconductor, with its *E*_f_ close to the CB. Upon contact, a *p-n* heterojunction is formed, resulting in the diffusion of electrons from the *n*-type V_2_O_5_ into the *p*-BiOI. This results in the accumulation of negative charge in the *p*-BiOI region near the junction (Scheme 1b). Simultaneously, holes migrate from the *p*-BiOI to the *n*-V_2_O_5_ region, resulting in the formation of a positively charged region in *n*-V_2_O_5_. The equilibration of the Fermi levels creates an internal electric field from *n*-V_2_O_5_ to *p*-BiOI, which prevents further charge diffusion, consistent with the literature [54,55].

Both the *p*-type BiOI and *n*-type V_2_O_5_ are excited by VL irradiation, generating electron–hole pairs. While rapid recombination typically limits photocatalytic efficiency, the well-aligned band structures and strong contact between BiOI and V_2_O_5_ promote effective charge separation. Electrons in the CB of *p*-BiOI efficiently migrate to the CB of *n*-V_2_O_5_, while positively charged centers, holes, move from the higher potential VB of V_2_O_5_ to the lower potential VB of *p*-BiOI. This charge transfer is facilitated by the close contact between the materials and the more negative CB edge potential of *p*-BiOI compared to *n*-V_2_O_5_, leading to improved photocatalytic efficiency.

The electric field formed at the interface further drives the transfer of photo-induced carriers, enhancing the separation of excitons and reducing recombination. The result is corroborated by photoluminescence and UV–visible absorption analysis, as displayed in Figure 7a and Figure S5. The accumulated electrons in the CB of V_2_O_5_ interact with adsorbed O_2_ on the surface of the BVNC to produce O_2_^•−^, which then oxidize organic pollutants into smaller molecules like CO_2_, H_2_O, and other in/organic compounds. Similarly, accumulation of the holes in the VB of BiOI oxidizes pollutants either directly or by reacting with OH^−^ or H_2_O to form hydroxyl radicals (OH^•^), which further degrade pollutants into CO_2_, H_2_O, and other in/organic molecules, which is further supported by the LC-MS analysis.

### 2.8. Detection of Degradation Fragments via LC-MS

To identify the photo-decomposed products in MB solutions after photocatalytic action by BVNC-1, LC-MS analysis was used. The control sample was analyzed prior to photodegradation, which showed the characteristic m/z intense peak at 283 (Figure S11a). The photocatalytic treatment of MB by BVNC-1 resulted in the formation of intermediates or byproducts, identifiable by specific m/z values (Figure S11b). These major m/z values were used to determine the structural formulas of the photodegradation products, as illustrated in Reaction Scheme S1. The LC-MS spectra revealed the formation of smaller organic compounds, H_2_O, CO_2_, nitrates, sulfates, and acetic acid as a result of the photodegradation process [48].

### 2.9. Bactericidal Activity

**Figure 9:**
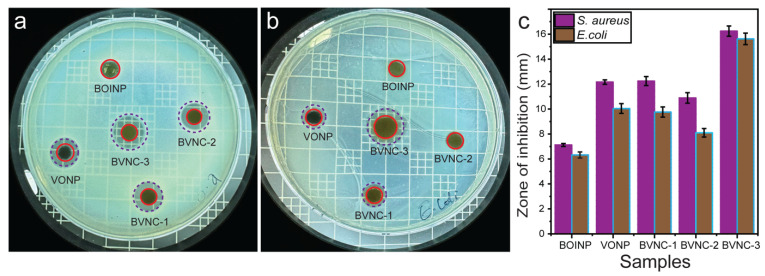
Digital photograph of antibacterial performance of nanocomposites and nanoparticles against *S. aureus* (**a**) and *E. coli* (**b**), and their corresponding zone of inhibition (**c**), respectively.

The antibacterial performance of the individual and composite materials to kill the *S. aureus, Gram-positive,* and the *E. coli,* Gram-negative, is shown in Figure 9 and Figure S12. The experiment was performed in triplicate, and the reported zones of inhibition (ZOI) values represent the average of these evaluations, as shown in Figure 9a–c. The results, achieved by utilizing the standard agar disk diffusion method, show that nanocomposite materials exhibited larger ZOI (Figure 9c) compared to the individual materials, confirming their excellent suitability for antibacterial performance.

The composite materials BVNC-1, BVNC-2, and BVNC-3 exhibit significantly stronger antibacterial activity for *E. coli* compared to BOINP and VONP when used individually. Similarly, the performance of the composite BVNP-3 demonstrated an excellent bactericidal effect on VONP. This enhanced antibacterial effect is attributed to the synergistic interactions of the hetero-composites in the BVNC series. Among these series of composites, BVNC-3 demonstrates the strongest antibacterial properties, likely due to its higher concentration of metal ions (V^5+^ and Bi^3+^) [20,56] and an increased formation of ROS, both of which are toxic to bacteria. However, BVNP-1 and -2 have similar activity with respect to each other. Interestingly, the inhibition zone for *S. aureus* was larger than that for *E. coli*, which could be due to the relatively more fragile or brittle cell wall of *S. aureus* [33,57], making it more susceptible to disruption by the as-synthesized materials.

The antibacterial properties of the nanocomposites can be attributed to both direct interactions with bacterial cells and the production of byproducts that aid in bacterial destruction. This enhanced performance results from two main mechanisms: the release of metal ions in high concentrations and the ROS under UV irradiation. The primary antibacterial action of the nanocomposites comes from their ability to release high concentrations of metal ions like V^5+^ and Bi^3+^. These metal ions interact with bacterial cell membranes, enhancing the antibacterial effect. The positively charged ions (V^5+^ and Bi^3+^) can penetrate the negatively charged bacterial membranes and interact with the sulfhydryl (−S-H) groups, weakening the bacteria, inhibiting growth, and ultimately leading to cell death [58]. Additionally, the generated ROS, including OH, O_2_^•−^, and H_2_O_2_, contribute to bacterial death [59,60,61]. ROS molecules penetrate bacterial membranes, disrupting biochemical processes and damaging cellular elements such as DNA, proteins, and lipids, which essentially results in bacterial death. Furthermore, the combined effect of metal ion release and ROS formation leads to potassium ion leakage (K^+^), further contributing to bacterial death.

## 3. Experimental Section

### 3.1. Chemical Reagents

Bismuth(III) nitrate pentahydrate (Bi(NO_3_)_3_·5H_2_O) (≥98%, Sigma-Aldrich, St. Louis, MO, USA, hereinafter “SA”), potassium iodide (KI, ≥99%, SA), ammonium metavanadate (ACS reagent, 99.95%, SA), ethyl alcohol (200 proof, ≥99.5%, SA), methylene blue (high purity, Alfa-Aesar, Ward Hill, MA, USA), Congo red (high purity, SA), rhodamine 6G (SA), methyl orange (SA), sodium chloride (NaCl, ≥99.0%, SA), sodium hydroxide (NaOH, reagent grade, ≥98.0%, SA), hydrochloric acid (HCl, SA), isopropyl alcohol (IPA, ≥99.7%, Fragrance Grade, SA), and benzoquinone (BQ, ≥98%, reagent grade, SA) were used as received.

### 3.2. Synthesis of BiOI/V_2_O_5_ Nanocomposites

**Scheme 2:**
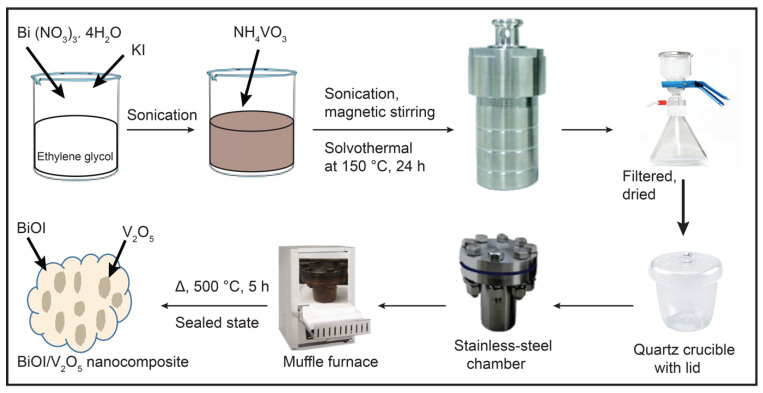
Schematic representation of the synthesis of BVNC.

The BVNC was synthesized using the solvothermal method followed by high-pressure annealing. Firstly, in 40 mL of ethylene glycol, 2.4253 g of bismuth nitrate pentahydrate was dissolved (Scheme 2) and followed by sonication for 30 min at room temperature. Secondly, 0.83 g of potassium iodide was poured into the solution, followed by additional sonication for half an hour to make the solution homogeneous until the reaction mixture turned pink due to the formation of the BiOI compound. Thirdly, 0.2 g of ammonium metavanadate was mixed, and the resulting solution was subjected to an additional 30 min of sonication. The sonicated mixture was agitated for 5 h at ambient temperature. Finally, the solution was placed into a Teflon-lined autoclave (100 mL), and the reaction chamber was kept in an oven at 150 °C for 24 h. Afterward, the mixture was filtered using a vacuum pump to separate the solid residue, which was then dried in a pre-heated oven at 70 °C for 10 h and retained in a quartz crucible with a lid. To prevent atmospheric oxygen from interfering with the reaction, the crucible was placed inside a stainless-steel chamber with a well-fitted (sealed) copper gasket (SUS314) to maintain a high pressure (ca. 2–3 atm.) inside the chamber. The chamber was subjected in a muffle furnace (Model-KSL-1100X-S-LD) and annealed at 500 °C for 5 h. The as-synthesized nanocomposite was rinsed with water and ethanol multiple times. The washed sample was dried in an oven at 70 °C for 10 h, named BVNC-1, and stored in an airtight vial for further analysis. For the synthesis of different composites, the same synthesis protocol was used with varying amounts of NH_4_VO_3_, resulting in BVNC-2 and BVNC-3, which contained 0.4 g and 0.6 g of NH_4_VO_3_, respectively.

### 3.3. Synthesis of BiOI Nanoparticles and V_2_O_5_ Nanoparticles

The VONP nanosheets and BOINP nanoflowers were synthesized using NH_4_VO_3_, Bi(NO_3_)_3_·5H_2_O, potassium iodide, and ethylene glycol, following the protocols described in Section 3.2. VONP and BOINP nanoparticles were used as control samples to determine the photocatalytic efficiency of the BiOI/V_2_O_5_ nanocomposite.

### 3.4. Characterization

The morphological and physicochemical properties of as-synthesized photocatalysts were thoroughly examined with the help of various techniques, as detailed in the Supplementary Materials.

### 3.5. Photocatalytic Degradation

The photocatalytic efficiency of the materials was assessed by monitoring the decomposition of MB dye under VL (300 W Xe lamp), using a UV cut-off filter (≥400 nm) to eliminate UV light interference. For each experiment, 50.00 mg of the photocatalyst was spread in 100 mL of a 15.00 mg/L MB dye solution in a 250 mL beaker. The suspension was agitated for 30 min to confirm efficient adsorption of the dyes onto the photocatalyst surface. Following this, for 30 min, the reaction mixture was stirred in the absence of light and left for one hour to maintain an equilibrium of adsorption–desorption. In exposing the mixture to VL radiation, the photocatalytic reaction was initiated, and the degradation of MB was observed over 140 min with the help of a UV-Vis spectrophotometer. Samples (3.5 mL) were withdrawn every 10 min for analysis, and the decomposition percentage was determined by recording the change in the dye’s absorbance at its characteristic wavelength (λ-max = 664 nm). Similarly, R6G, MO, and BPA degradation was analyzed at their respective λ-max values: 525 nm for R6G, 464 nm for MO, and 275 nm for BPA. All photocatalytic experiments were conducted under identical conditions. Additionally, a control experiment was conducted using only the MB dye solution without the photocatalyst to confirm the absence of degradation under VL. The photocatalytic efficiency (%) was calculated using Equation (5).

(5) $$\eta \left(\%\right)=\frac{C_{0}-C_{t}}{C_{0}}\times 100$$

where *C*_0_ is the concentration of the pollutant at time zero, *C*_t_ is the concentration of the pollutant at illumination time period *t*, and *η* is the degradation efficiency. Similarly, Equation (6) was employed to determine the rate of reaction (*k*) for the photodegradation:

(6) $$k=\ln\frac{C_{0}∕C_{t}}{t}$$

### 3.6. Reusability Assessment

The recyclability of the best-performing photocatalyst was analyzed using a standardized protocol. After each test, the residue was centrifuged, rinsed, and dried at 80 °C for 8 h. Consistent experimental conditions were maintained throughout the testing, ensuring the reliability and validity of the results obtained.

### 3.7. Deduction of the Point of Zero Charge and pH Effects

The pH drift method was used to analyze the point of zero charge (pH_pzc) of the BVNC. A 100 mL beaker containing 0.01 M NaCl was prepared to obtain the desired initial pH using 0.01 M NaOH or 0.01 M HCl. The pH-adjusted NaCl (20 mL) was then transferred to a vial containing 10.0 mg of BVNC photocatalyst. The final solution was stirred for 24 h to disperse the photocatalyst. Initial pH values (2, 4, 6, 8, 10, and 12) were used, and the corresponding final pH values were noted. The pH_pzc value was calculated by plotting the variation in pH (∆pH) over the initial pH (pH_i).

### 3.8. Charge Trapping Experiments

Charge trapping experiments were performed using the optimal catalyst, BVNC, to understand the role of carriers in the MB decomposition in the presence of VL irradiation. Isopropyl alcohol (3 mL), 1,4-benzoquinone (5.0 mg), and potassium iodide (5.0 mg) were employed to selectively scavenge hydroxyl (OH^•^) radicals, holes, and superoxide radicals (O_2_^•−^), respectively. Each scavenger was put into the MB solution encompassing the photocatalyst, and the photodegradation experiment was carried out as described in Section 2.6.

### 3.9. Liquid Chromatography-Mass Spectroscopy (LC-MS) Analysis

LC-MS investigation was performed before and after photocatalytic treatment to identify the degradation products of MB in the presence of VL. An aliquot (5 μL) of each sample was inserted into the LC system (LC 1200 Series) for analysis. Electrospray ionization in positive mode was used to generate ions, and standard operating conditions were applied for the analysis.

### 3.10. Antibacterial Activity

The antibacterial properties of both individual and synthesized materials were assessed by observing the ZOI produced by various nanocomposites against resistant strains of *Staphylococcus aureus* (*S. aureus*, ATCC 29231) and *Escherichia coli* (*E. coli*, ATCC 52922). Tryptic soy broth was used to culture the bacteria, and incubation was performed at 37 °C for 12 h with agitation at 100 rpm in a shaking incubator. Before incubation, a suspension of bacteria containing 1 × 10^7^ colony-forming units was evenly distributed onto agar Petri dishes [33]. After 12 h of exposure to UV light, the nanocomposites were thoroughly cleaned and used to create a colloidal solution. Each nanocomposite, having a concentration of 10 mg/mL, was dispersed in 500 μL of deionized water. The resulting colloidal suspensions were then applied to the bacteria-laden Petri dishes and incubated at 37 °C for another 12 h to assess their antibacterial activity. The bactericidal effectiveness of as-synthesized nanocomposites was assessed by measuring the ZOI, which was photographed and studied digitally.

## 4. Conclusions

BiOI/V_2_O_5_ heterojunction photocatalysts were successfully synthesized through a solvothermal process followed by high-pressure annealing. Morphological analysis revealed the formation of VONP, which adhered to flower-like BOINP structures, resulting in unique hetero-composites. The composite materials demonstrated superior photocatalytic performance compared to the individual BOINP and VONP components. Among the synthesized nanocomposites, the best-performing BVNC-1 exhibited the highest photodegradation activity, achieving 95.7% decomposition of MB, 94.0% of R6G, 91.4% of MO, and 69.5% of BPA under VL irradiation. A scavenger test identified O_2_^•−^ as a key active species responsible for the degradation of MB, and LC-MS analysis confirmed the breakdown of MB into smaller fragments. The nanocomposites also showed excellent recyclability and stability, demonstrating a retention of catalytic activity of 81.8% until the fourth cycle. Similarly, the BVNC-3 showed excellent antibacterial activity, having a ZOI of 12.24 and 11.62 nm against *S. aureus* and *E. coli*, respectively. The enhanced photocatalytic and antibacterial performance was attributed to the efficient carrier transport across the *p-n* heterojunction of the composites. These results may provide valuable insights into the strategic design of Bi-derived nanocomposites for effective environmental remediation and antibacterial applications.

## Data Availability

Data are contained within the article or Supplementary Materials.

## Supplementary Materials

The following supporting information can be downloaded at: https://www.mdpi.com/article/10.3390/molecules30122500/s1, References [62,63,64,65,66,67,68,69,70] are cited in Supplementary Materials. Figure S1: EDS elemental analysis of BVNC-1: (a) FE-SEM image; (b) EDS layered image of (a), with O, I, Bi, and V represented by green, dark blue, light blue, and orange, respectively; in the nanocomposite, (c) EDS spectrum of the entire region shown in (a). Elemental mapping of individual elements of (d) O, (e) I, (f) Bi, and (g) V. Table S1: Theoretical and experimental elemental weight and atomic percentage in the BVNC-1. Figure S2: Raman Spectroscopy of (a) VONP, (b) BIONP, (c) BVNC-1, (d) BVNC-2, and (e) BVNC-3, respectively. Figure S3: XPS survey spectra of BOINP, VONP, and BVNC composites. Figure S4: Photoluminescence characterization of various nanoparticles and nanocomposites. Figure S5: UV-Vis spectra of individual nanoparticles and nanocomposites: (a) BVNC-1, BVNC-2, (c) BVNC-3, (d) BOINP, (e) VONP, and (f) Blank test of MB. Figure S6: The kinetic plot, R^2^ values, and rate constant values R6G (a, b), MO (c, d), and BPA (e, f). Table S2: R^2^ value of pseudo-first-order reaction of B/V related materials. Figure S7: (a) Effect of catalyst dose (BVNC-1) for MB degradation, (b) Effect of MB pollutant concentration using 50.0 mg of photocatalyst (BVNC-1). Figure S8. Determination of the point of zero charge of the BVNC-1. Figure S9. pH-dependent study of MB and MO at pH, 4,7, and 10. The experiment was conducted with the aid of a light source (model name: Thorlabs SOLIS-3C M00909170) system, where the solution was positioned 25 cm away from the light source. Figure S10: Charge carriers trapping experiments of MB using BVNC-1 photocatalyst. These experiments reveal that O_2_^•−^ are the most dominant contributor to the photocatalytic decomposition of MB, with h^+^ and active OH^•^ also playing significant roles in the reaction. Figure S11: LC-MS spectra of the MB solution; (a) before undergoing the photocatalytic decomposition, and (b) after the photocatalytic action of BVNC-1 photocatalyst. Reaction Scheme S1. Possible degradation pathway of MB. Table S3: Comparison of the photocatalytic efficiency of BiOI/V_2_O_5_ composite and related composite materials. Figure S12: The determination of the ZOI Via measuring the diameter.
