# Supplementary material for: Harnessing BiOI/V2O5 Nanocomposites: Advanced Bifunctional Catalysts for Visible-Light Driven Environmental Remediation and Antibacterial Activity
Source: Molecules. 2025 Jun 6;30(12):2500. doi: 10.3390/molecules30122500 (PMC12196363; doi:10.3390/molecules30122500)
Supplement: Supplementary file 1 [file molecules-30-02500-s001.zip › molecules-3629411-supplementary.pdf]

Harnessing BiOI/V_2O_5 Nanocomposites: Advanced Bifunctional Catalysts for Visible-Light Driven Environmental Remediation and Antibacterial Activity

Anil Pandey[a,+], Narayan Gyawali[a,+], Devendra Shrestha[b], Insup Lee[a], Santu Shrestha[a], Subas Acharya[a], Pujan Nepal[a], Binod Gaire[a], Vince Fualo[a], Sabita Devi Sharma[c], Jae Ryang Hahn[a,d,*]

[a]*Department of Chemistry, Jeonbuk National University, Jeonju, 54896, Korea*

[b]*Department of Bionanotechnology and Bioconvergence Engineering, Graduate School, Jeonbuk National University, Jeonju 561-756, Republic of Korea*

[c]*Department of Physics, Birendra Multiple Campus, Chitwan, 44207, Nepal.*

[d]*Textile Engineering, Chemistry and Science, North Carolina State University 2401 Research Dr., Raleigh, North Carolina 27695-8301, United States*

[+]*AP and NG contributed equally to this paper.*

**Corresponding author*

S1. Characterization techniques

The morphology of the synthesized samples was scrutinized using high-resolution scanning electron microscopy (HR-SEM, SU8230, Hitachi, Japan) and High-resolution transmission electron microscopy (HR-TEM-2200FS, JEOL, Japan). To assess the elemental composition and purity of the composite, energy-dispersive spectroscopy (EDS) was used. Fourier transform infrared (FTIR) spectroscopy (Thermo Fischer Scientific, Nicolet iS5) was applied to investigate the functional groups of the composites, with pellets of the nanocomposites prepared using potassium bromide and used for the analysis. For a comprehensive understanding of the binding energies, oxidation state, and chemical composition of the various components in the prepared composites, X-ray photoelectron spectroscopy (XPS, NEXSA, Thermo Fisher Scientific, UK). X-ray spectroscopy (EDS) was employed. A high-resolution X-ray diffractometer (X'Pert PRO, PANalytical, Netherlands) equipped with a Cu Ka (λ=1.5406 A°) radiation source was used to analyze phase composition and crystal structure. Raman spectroscopy was performed with the help of high-performance 3D imaging Raman (Nano photon-Raman Touch). The samples' optical absorption properties and bandgaps were evaluated using a UV-vis spectrophotometer (Perkin Elmer Lambda 25, Ayer Rajah, and Singapore). Photoluminescence (PL) emission spectra were recorded at room temperature with a spectrofluorometer (FP-8350, Jasco) using an excitation wavelength of 370 nm. Liquid chromatography-mass spectroscopy (LC-MS, Agilent 6410B, and Wilmington, USA) was employed to analyze aqueous solutions of methylene blue (MB) after 140 minutes of photocatalysis, aiming to investigate the degradation products of the MB dye.

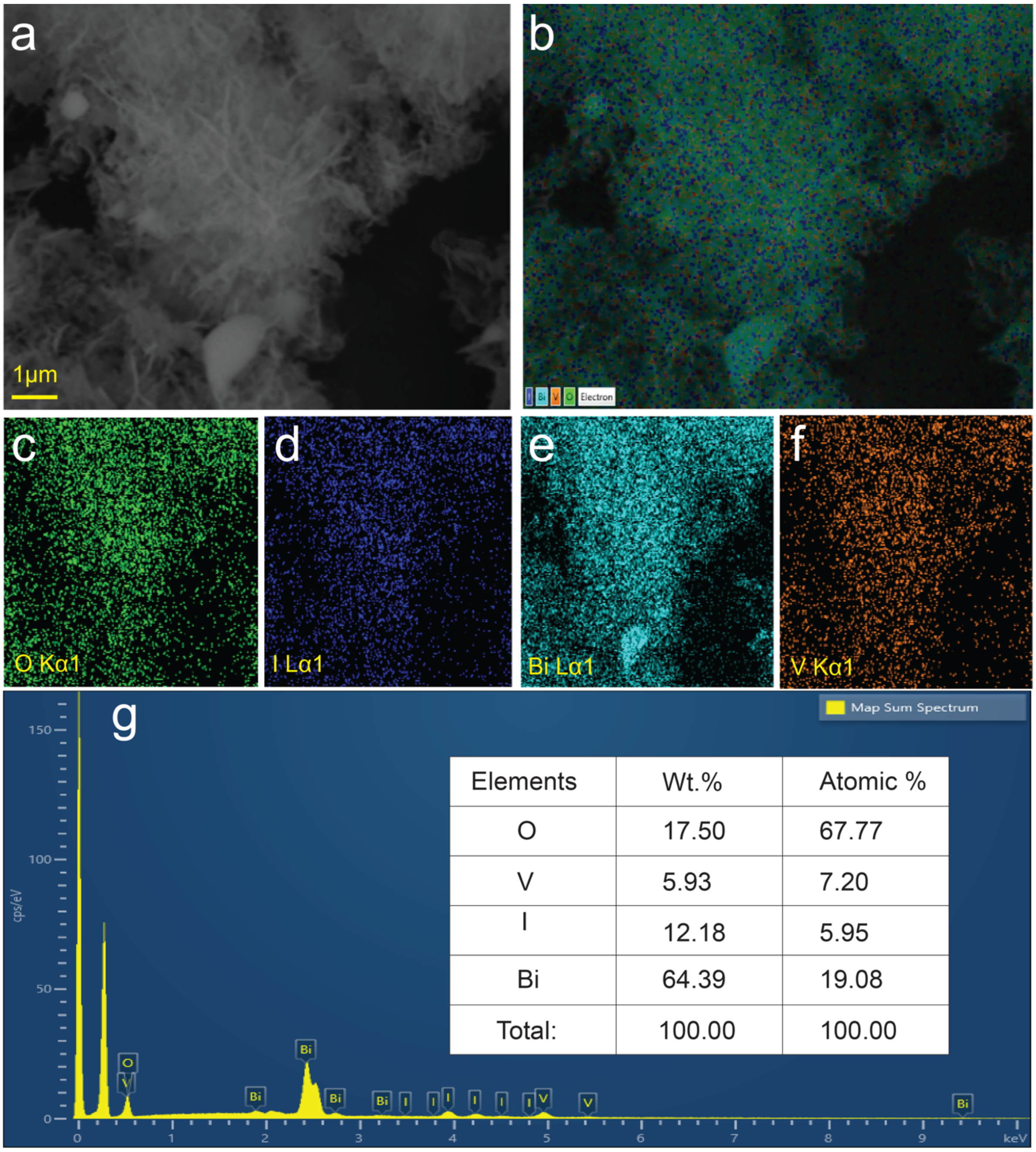

Elements	Wt.%	Atomic %
O	17.50	67.77
V	5.93	7.20
I	12.18	5.95
Bi	64.39	19.08
Total:	100.00	100.00

Figure S1. EDS elemental analysis of BVNC-1: (a) FE-SEM image; (b) EDS layered image of (a), with O, I, Bi, and V represented by green, dark blue, light blue, and orange respectively; in the nanocomposite, Elemental color mapping of individual elements of (c) O, (d) I, (e) Bi, and (f) V, and (g) EDS spectrum, respectively.

Table S1. Theoretical and experimental elemental weight and atomic percentage in the BVNC-1.

Theoretical Calculation			Experimental from EDS analysis		
Element	Weight %	Atomic %	Element	Weight %	Atomic %
Bi	54.6	23.8	Bi	64.39	19.08
I	33.1	23.8	I	12.18	5.95
V	4.6	8.1	V	5.93	7.20
O	7.7	44.3	O	17.50	67.77
Total	100	100		100	100

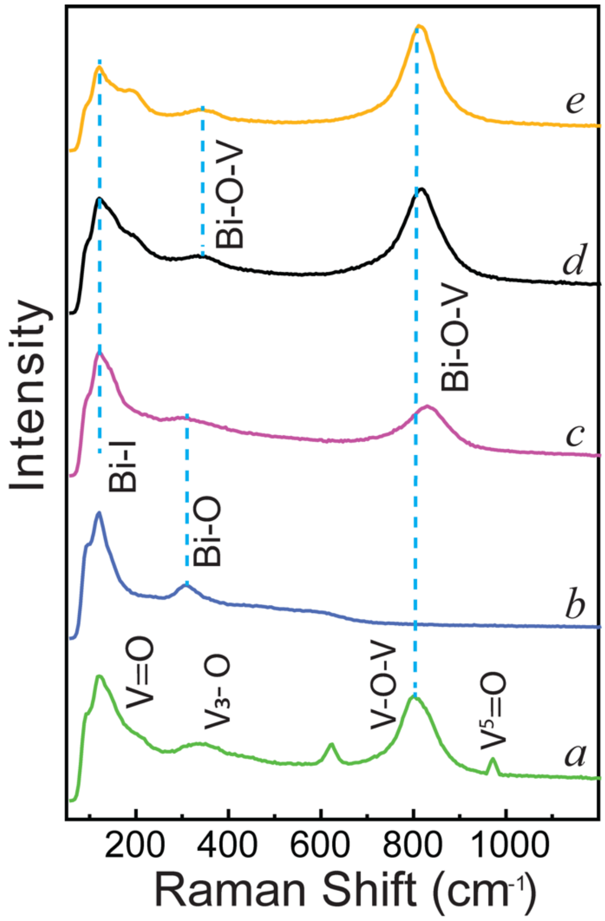

Figure S2. Raman Spectroscopy of (a) VONP, (b) BIONP, (c) BVNC-1, (d) BVNC-2 and (e) BVNC-3, respectively.

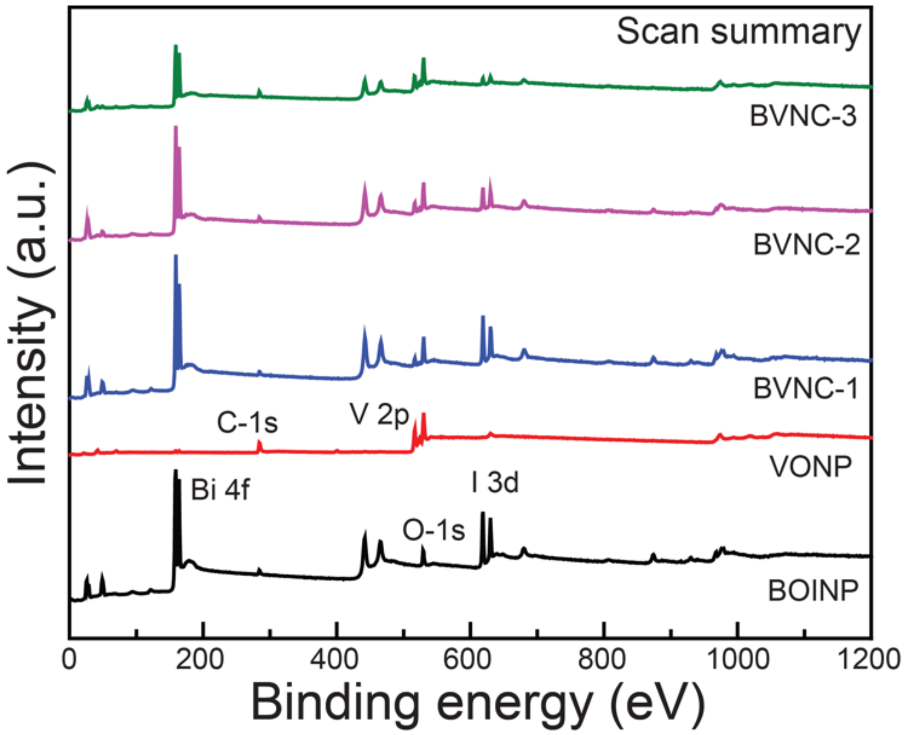

Figure S3. XPS survey spectra of BOINP, VONP, and BVNC composites.

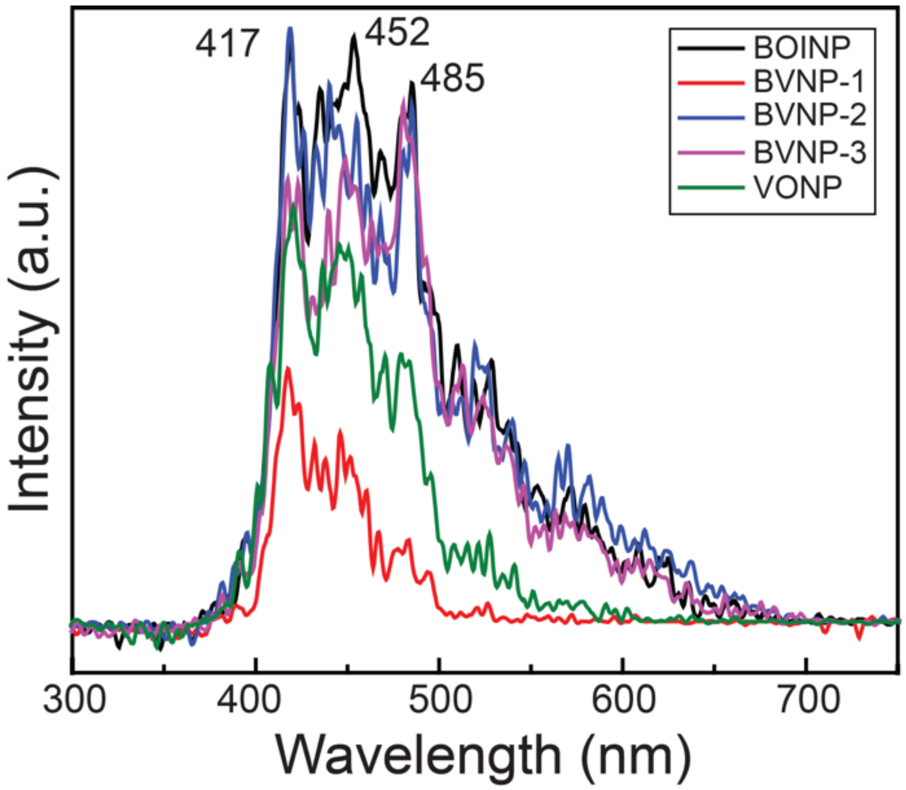

Figure S4. Photoluminescence characterization of various nanoparticles and nanocomposites.

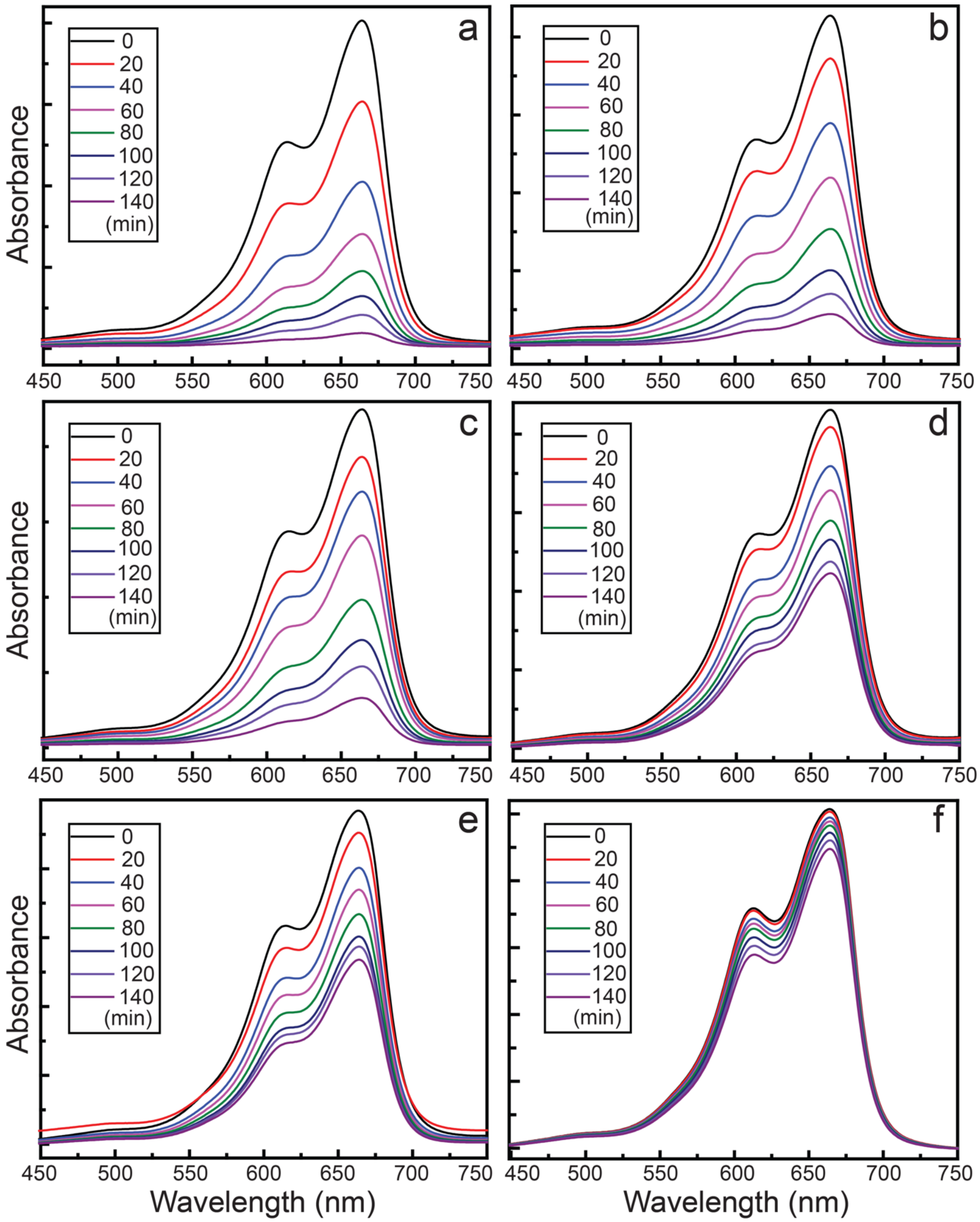

Figure S5. UV-Vis spectra of individual nanoparticles and nanocomposites: (a) BVNC-1, BVNC-2, (c) BVNC-3, (d) BOINP, (e) VONP, and (f) Blank test of MB, respectively.

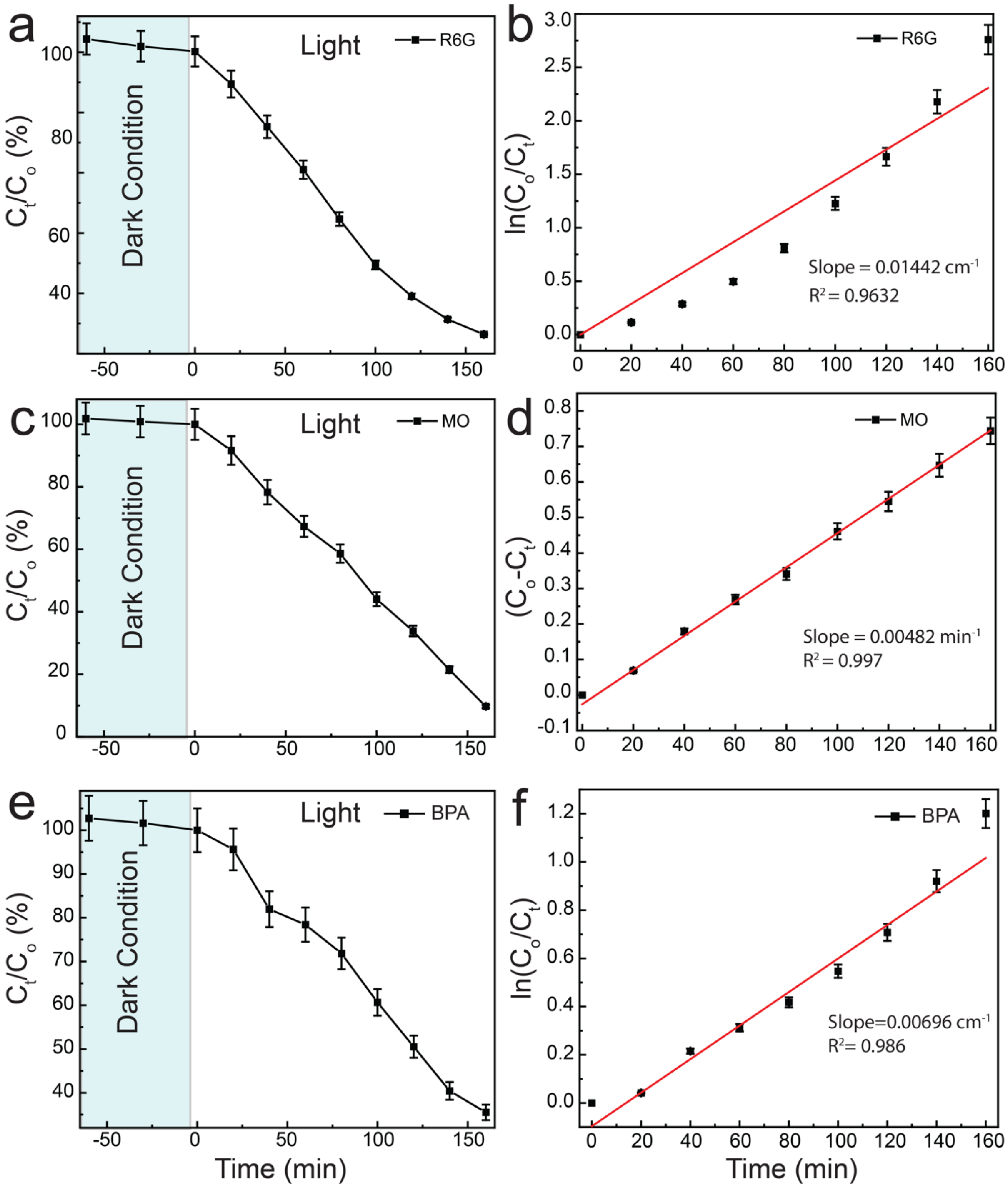

Figure S6. Degradation vs time plot and kinetics plots of R6G (a, b), MO (c, d), and BPA (e, f), respectively.

Table S2. R^2 value of pseudo-first-order reaction of B/V related materials.

Catalysts	MB	BOINP	VONP	BVNC-1	BVNC-2	BVNC-3
R-Square	0.95119	0.9842	0.99621	0.9808	0.97862	0.9245

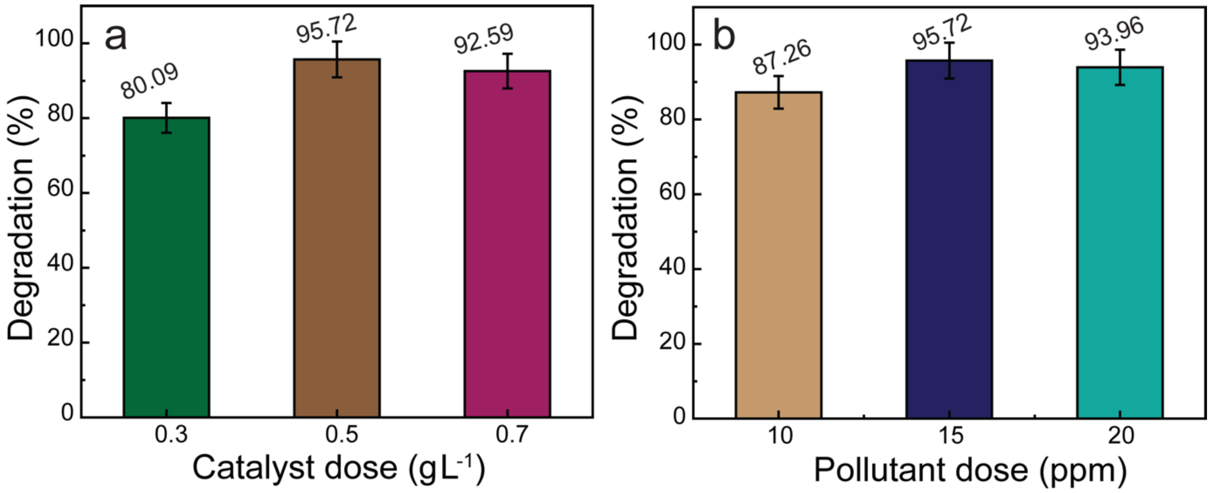

Figure S7. (a) Effect of catalyst dose (BVNC-1) for MB degradation, (b) Effect of MB pollutant concentration using 50.0 mg of photocatalyst (BVNC-1).

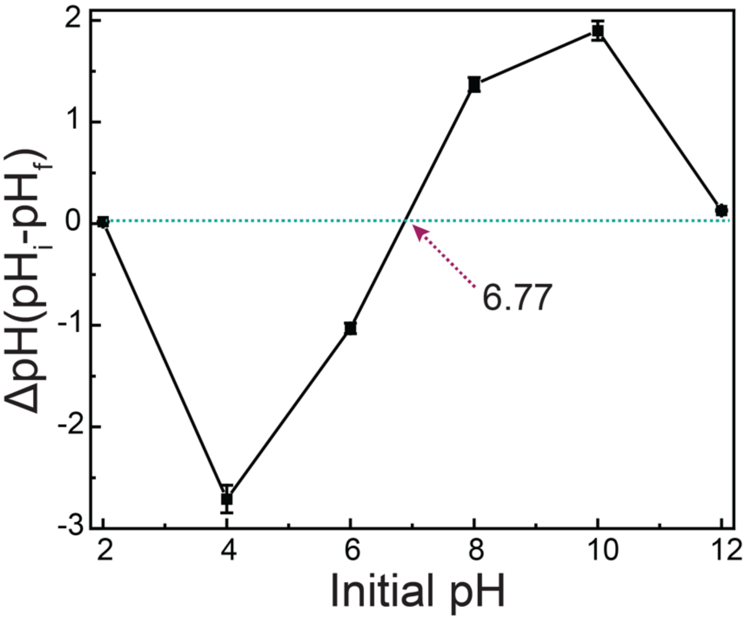

Figure S8. Determination of the point of zero charge of the BVNC-1.

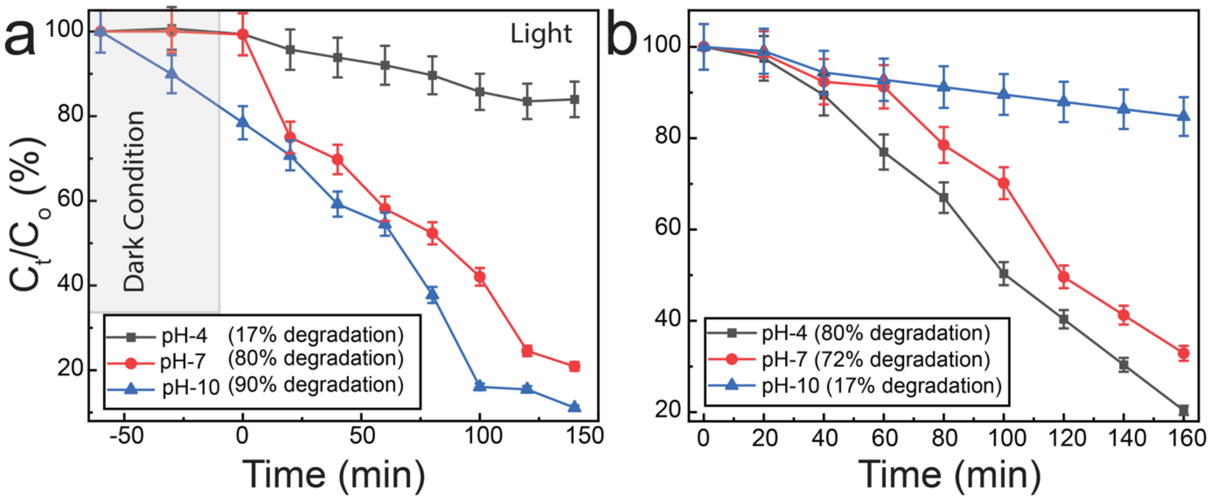

Figure S9. pH dependent study of MB and MO at pH of 4,7, and 10. The experiment was conducted with the help of light source (model name: THORLABS SOLIS-3C M00909170) system where solution was kept 25 cm far from light source.

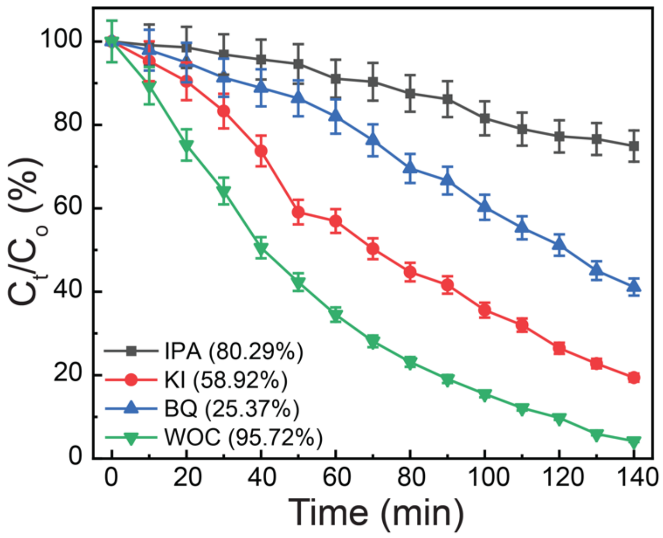

Figure S10. Charge carriers trapping experiments of MB using BVNC-1 photocatalyst. These experiments reveal that $O_2^{\bullet -}$ radicals are the most dominant contributor to the photocatalytic degradation of MB, with h^+ and active $OH^{\bullet}$ also playing significant roles in the reaction.

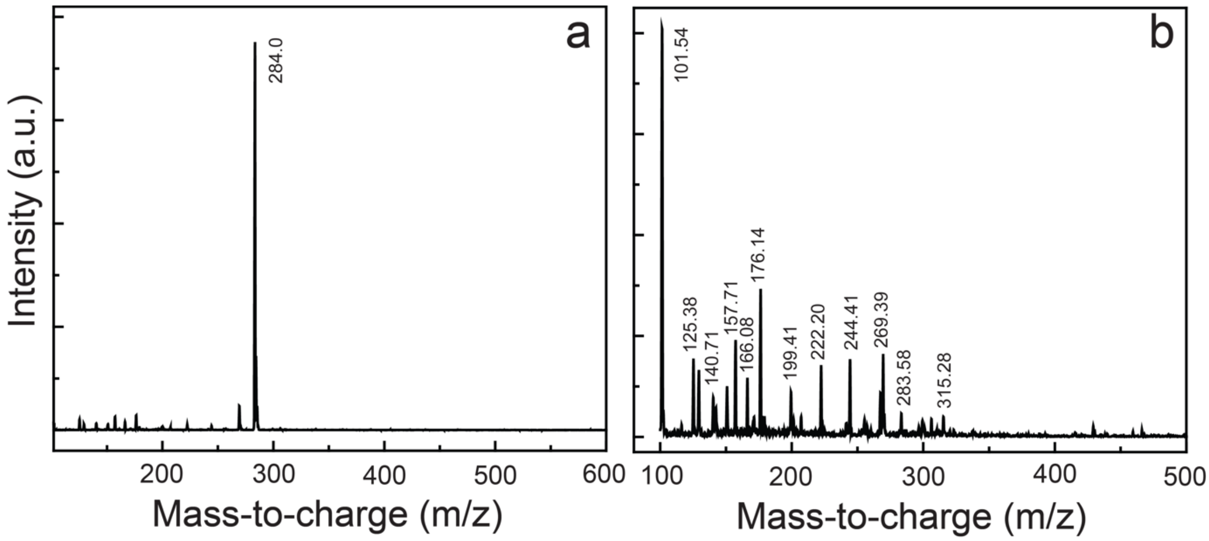

Figure S11. LC-MS spectra of the MB solution; (a) before undergoing the photocatalytic decomposition, and (b) after the photocatalytic action of BVNC-1 photocatalyst.

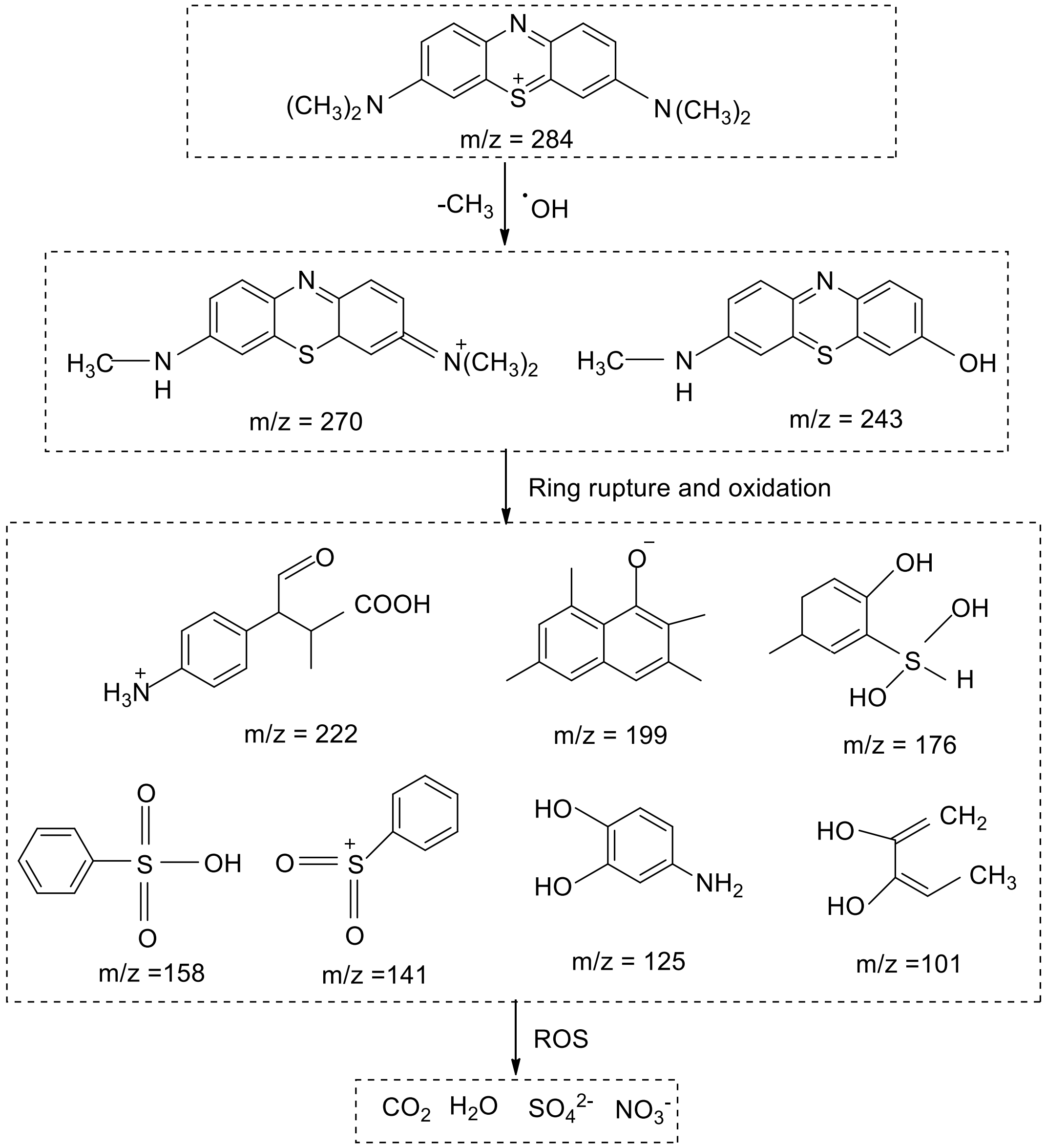

Reaction Scheme RS1. Possible degradation pathway of MB.

Table S3. Comparison of the photocatalytic efficiency of BiOI/V_2O_5 composite and related composite materials.

Composite	Pollutant	Pollutant concentration	Composite dose (100 mL)	Degrad-ation (%)	Light source /Degradation time	References
V_2O_5 NPs	R6G	24 mg/L	20 mg	85	VL/300 min	[1]
V_2O_5 NPs	MB	3.2 mg/L	20 mg	40	300 W LED light/180 min	[2]
p-BiOI/ZnTiO	R6G	50 mg/L	100 mg	82	UV/180 min	[3]
V_2O_5/TiO_2	RhB	4 mg/L	2 mg	94	500 W halogen lamp, VL /6h	[4]
BiOI/ZnO	MB	20 mg/L	50 mg	94	300W Xe lamp/180 min	[5]
Fe_3O_4/BiOI	RhB	100 mg/L	NA	100	330W Xe lamp/240 min	[6]
g-C_3N_4/BiOI	MB	20 mg/L	150 mg	92	VL /180 min	[7]
V_2O_5/CdS	CR	20 mg/L	2 mg	77	VL /5h	[8]
V_2O_5/rGO	MB	10 mg/L	10 mg	82	UVL/300 min	[9]
CeO_2/V_2O_5	MB	10 mg/L	5 mg	64	VL /210 min	[10]
BiOI/V_2O_5	MB<br>R6G<br>MO<br>BPA	15 mg/L	50 mg	96<br>94<br>90<br>70	VL /<br>140 min<br>160 min<br>160 min<br>160 min	**This study**

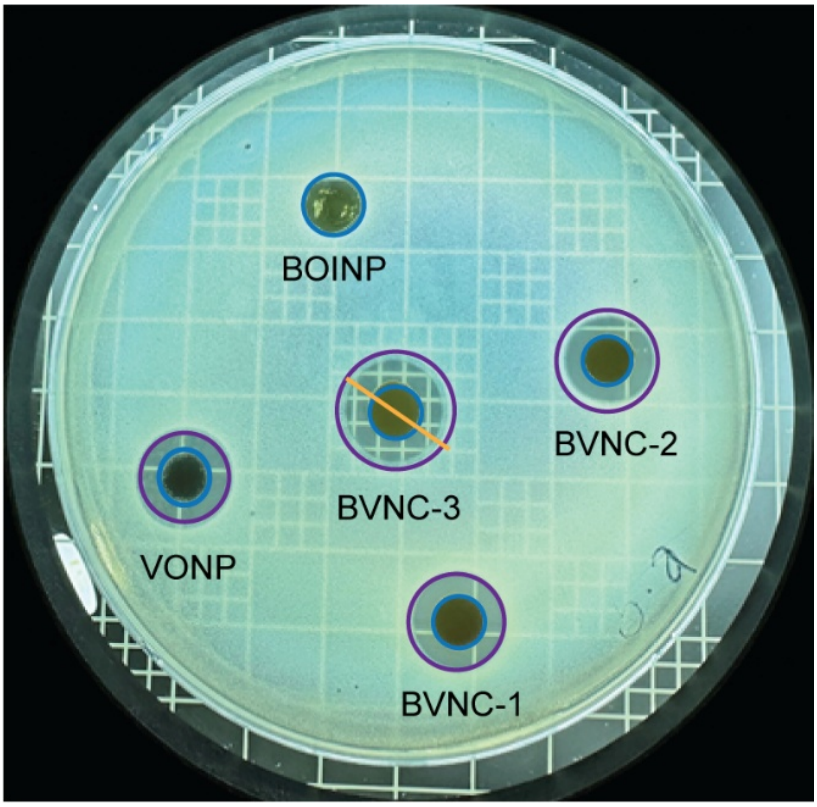

Figure S12. The determination of the ZOI via measuring the diameter.

References

[1] S. K. Jayaraj, V. Sadishkumar, T. Arun, P. Thangadurai, Enhanced photocatalytic activity of V_2O_5 nanorods for the photodegradation of organic dyes: A detailed understanding of the mechanism and their antibacterial activity, *Mater. Sci. Semicond. Process.*, 85 (2018) 122-133. DOI: https://doi.org/10.1016/j.mssp.2018.06.006

[2] M. Jalil, M. Khan, S. Mandal, F. U. Z. Chowdhury, M. Hossain, D. Jana, M. Alam, M. Uddin, Impact of reaction temperatures on the particle size of V_2O_5 synthesized by facile hydrothermal technique and photocatalytic efficacy in dye degradation, *AIP Adv.*, 13 (2023). DOI: https://doi.org/10.1063/5.0125200

[3] K. H. Reddy, S. Martha, K. Parida, Fabrication of novel p-BiOI/n-$ZnTiO_3$ heterojunction for degradation of rhodamine 6G under visible light irradiation, *Inorg. Chem.*, 52 (2013) 6390-6401. DOI: https://doi.org/10.1021/ic400159m

[4] M. Mondal, H. Dutta, S. Pradhan, Enhanced photocatalysis performance of mechano-synthesized V_2O_5-TiO_2 nanocomposite for wastewater treatment: correlation of structure with photocatalytic performance, *Mater. Chem. Phys.*, 248 (2020) 122947. DOI: https://doi.org/10.1016/j.matchemphys.2020.122947

[5] W. Xiao, Y. Su, J. Luo, L. Jiang, X. Wu, Z. Liu, H. Pang, Q. Zhang, P. Zhang, Flower-like hierarchical architecture of BiOI/ZnO pn junction composites with high-efficient visible-light photodegradation activities, *Solid State Sci.*, 108 (2020) 106432. DOI: https://doi.org/10.1016/j.solidstatesciences.2020.106432

[6] H. Y. Xu, W. S. Wang, B. Li, L. Zhang, Mechanism insights into the enhanced photocatalytic peroxydisulfate activation by Fe_3O_4/BiOI heterojunction, *Mater. Sci. Eng. B*, 294 (2023) 116509. DOI: https://doi.org/10.1016/j.mseb.2023.116509

[7] J. Zhang, J. Fu, Z. Wang, B. Cheng, K. Dai, W. Ho, Direct Z-scheme porous g-C_3N_4/BiOI heterojunction for enhanced visible-light photocatalytic activity, *J. Alloys Compd*, 766 (2018) 841-850. DOI: https://doi.org/10.1016/j.jallcom.2018.07.041

[8] S. N. N. M. Makhtar, N. Yusof, N. Fajrina, N. H. H. Hairom, F. Aziz, W. N. Wan Salleh, V_2O_5/CdS as nanocomposite catalyst for Congo red dye photocatalytic degradation under visible light, *Mater. Today Proc.*, 96 (2024) 69-72. DOI: https://doi.org/10.1016/j.matpr.2023.10.152

[9] D. J. Ahirrao, S. N. Tambat, S. S. Sonawane, S. T. Alone, R. D. Pathrikar, V_2O_5 Based Nanocomposites for the Adsorption and Photocatalytic Degradation of Methylene Blue Dye, *Optik,* (2024) 171732. DOI: https://doi.org/10.1016/j.ijleo.2024.171732

[10] R. Saravanan, S. Joicy, V. K. Gupta, V. Narayanan, A. Stephen, Visible light-induced degradation of methylene blue using CeO_2/V_2O_5 and CeO_2/CuO catalysts, *Mater. Sci. Eng. C*, 33 (2013) 4725-4731. DOI: https://doi.org/10.1016/j.msec.2013.07.034
